# HNF4alpha and HNF1alpha Dysfunction as a Molecular Rational for Cyclosporine Induced Posttransplantation Diabetes Mellitus

**DOI:** 10.1371/journal.pone.0004662

**Published:** 2009-03-02

**Authors:** Jürgen Borlak, Monika Niehof

**Affiliations:** 1 Fraunhofer Institute of Toxicology and Experimental Medicine, Medical School of Hannover, Hannover, Germany; 2 Center of Molecular Medicine and Medical Biotechnology and Center of Pharmacology and Toxicology, Medical School of Hannover, Hannover, Germany; East Carolina University, United States of America

## Abstract

Posttransplantation diabetes mellitus (PTDM) is a frequent complication in immunosuppressive therapy. To better understand the molecular events associated with PTDM we investigated the effect of cyclosporine on expression and activity of hepatic nuclear factor (HNF)1alpha and 4alpha and on genes coding for glucose metabolism in cultures of the rat insulinoma cell line INS-1E, the human epithelial cell line Caco-2 and with Zucker diabetic fatty (ZDF) rats. In the pancreas of untreated but diabetic animals expression of HNF4alpha, insulin1, insulin2 and of phosphoenolpyruvate carboxykinase was significantly repressed. Furthermore, cyclosporine treatment of the insulinoma-1E cell line resulted in remarkable reduction in HNF4alpha protein and INS1 as well as INS2 gene expression, while transcript expression of HNF4alpha, apolipoprotein C2, glycerolkinase, pyruvatekinase and aldolase B was repressed in treated Caco-2 cells. Furthermore, with nuclear extracts of cyclosporine treated cell lines protein expression and DNA binding activity of hepatic nuclear factors was significantly repressed. As cyclosporine inhibits the calcineurin dependent dephosphorylation of nuclear factor of activated T-cells (NFAT) we also searched for binding sites for NFAT in the pancreas specific P2 promoter of HNF4alpha. Notably, we observed repressed NFAT binding to a novel DNA binding site in the P2 promoter of HNF4alpha. Thus, cyclosporine caused inhibition of DNA binding of two important regulators for insulin signaling, i.e. NFAT and HNF4alpha. We further investigated HNF4alpha transcript expression and observed >200-fold differences in abundance in n = 14 patients. Such variability in expression might help to identify individuals at risk for developing PTDM. We propose cyclosporine to repress HNF4alpha gene and protein expression, DNA-binding to targeted promoters and subsequent regulation of genes coding for glucose metabolism and of pancreatic beta-cell function.

## Introduction

In organ transplantation there is a need to suppress an immune response against the grafted organ. Immunosuppressive therapies with calcineurin inhibitors result, however, in unwanted secondary effects. This includes risk of infections of all types, lymphomas and other malignancies [1], [2]. Posttransplantion diabetes mellitus (PTDM) is a further complication with an incidence of approximately 8–10% for cyclosporine and 16–18% for tacrolimus across renal, liver, heart and lung transplant patients [3], [4]. Noteworthy, the DIRECT study reports a 36% incidence of impaired glucose metabolism and a 14% incidence of PTDM with either cyclosporine or tacrolimus [5]. Indeed, cyclosporine caused morphologic and functional alterations of pancreatic beta-cells with subsequent hyperglycemia and hypoinsulinoma in diverse animal studies [6]–[11]. Based on their mode of action cyclosporine and tacrolimus repress interleukin-2, thereby suppressing the early cellular response of T-lymphocytes to an antigenic stimuli. As of today the causes for the diabetogenic potential of calcineurin inhibitors remain uncertain. To better understand the molecular events associated with PTDM we investigated expression and activity of hepatic nuclear factor 1α (HNF1α) and 4α (HNF4α). Notably, dysfunction of these transcription factors have been associated with diabetes mellitus. For instance, the early onset of type II diabetes referred to as MODY (maturity onset diabetes of the young) was mapped to mutations within the *HNF1α* (MODY3) and *HNF4α* (MODY1) gene [12]. Moreover, linkage analysis in combination with fine-mapping for susceptibility to multifactorial late-onset type 2 diabetes has identified predisposing variants of *HNF4α* and *HNF1α* in a growing number of studies [13]–[15]. The HNF4α-dependent transcription of *HNF1α* is required for normal β-cell function [16], but there is also a feedback loop of HNF4α and HNF1α to maintain tissue specific metabolic function [16]–[18]. Additionally, in conditional *HNF4α* knockout mice β-cell function was impaired upon glucose-stimulated insulin secretion [19]–[21] whereas *HNF1α* knockout mice develop diabetes [22].

Taken collectively, HNF1α and HNF4α regulate various members of the glucose-dependent insulin secretory pathways [19]–[28] and might therefore provide a molecular rational for calcineurin inhibitor induced diabetes.

## Results and Discussion

**Figure 1:**
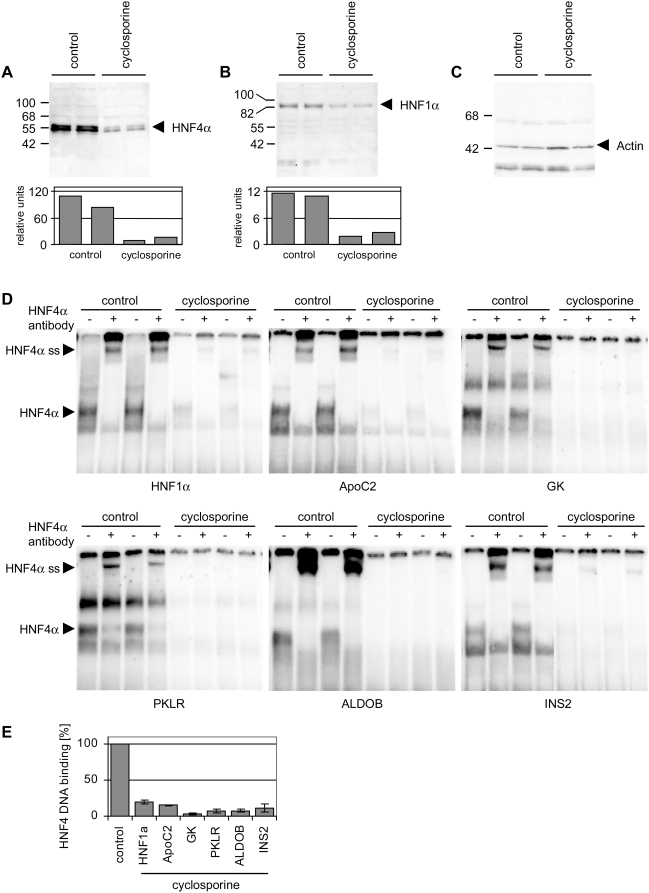
Cyclosporine inhibits protein expression of HNF4α and binding to target gene promoters. (A) HNF4α western blotting of 20 µg Caco-2 cell nuclear extracts [control or cyclosporine treatment, 25 µM (30 µg/ml) for 72 h]. (B) HNF1α western blotting of 30 µg Caco-2 cell nuclear extracts [control or cyclosporine treatment, 25 µM (30 µg/ml)for 72 h] (C) Actin western blotting of 15 µg Caco-2 cell nuclear extracts [control or cyclosporine treatment, 25 µM (30 µg/ml) for 72 h]. The lower panels represent the quantification of protein amounts for HNF4α (A) and HNF1α (B) relative to the actin expression. (D) Electrophoretic mobility shift assays with 2,5 µg Caco-2 cell nuclear extract [control or cyclosporine treatment, 25 µM (30 µg/ml) for 72 h] and ^32^P labeled oligonucleotides to probe for DNA binding to HNF4α binding-sites within promoters of HNF1α (HNF1α), apolipoprotein C2 (ApoC2), glycerol kinase (GK), pyruvate kinase (PKLR), aldolase B (ALDOB), and insulin2 (INS2). In EMSA supershift assays an antibody directed against HNF4α (+) was added. Shifted (HNF4α) and supershifted bands (HNF4α ss) were marked. (E) Dried EMSA gels were analyzed with a Molecular Imager (BioRad, Muenchen, Germany) using the Quantity One software (BioRad, Muenchen, Germany). HNF4α binding of control extracts to the respective binding sites was set to 100% and inhibition of binding to the respective binding sites after treatment with cyclosporine [25 µM (30 µg/ml) for 72 h] was quantified.

Initially, we investigated expression of *HNF4α* in the pancreas of Zucker diabetic fatty (ZDF) rats. This is an established disease model for type 2 diabetes. We observed reduced expression of *HNF4α* and of genes regulated by this factor in the glucose metabolic pathway, notably phosphoenolpyruvate carboxykinase 1 (*PCK1*), insulin1 (*INS1*) and insulin2 (*INS2*) (Table 1). Furthermore, HNF4α and HNF1α was significantly reduced in the liver of these animals (Table 1). In the past HNF4α was shown to regulate *INS1* [29]. As rodents express two isoforms of insulin (*INS1* and *INS2*) [30] both genes were investigated, but the physiological role of INS2 is not clear as yet [30]. By use of advanced bioinformatics we identified a new HNF4α binding site in the promoter of the INS2 gene at position −245 to −232 upstream of the start site of transcription [see Material and Methods for sequence information and electrophoretic mobility shift (EMSA) assay in Fig. 1D]. Loss of HNF4α DNA-binding to targeted promoters resulted in reduced expression of genes coding for glucose transport and metabolism and of insulin secretion from pancreatic ß-cells [28]. Furthermore, in conditional *HNF4α* knockout mice β-cell function was impaired upon glucose-stimulated insulin secretion [19]–[21]. Conversely, in HNF1α overexpressing beta cell lines increased transcript expression of insulin, glucose transporter 2, L-pyruvate kinase, and aldolase B was observed [26], [27] whereas *HNF1α* knockout mice developed diabetes [22].

**Table 1:** Regulation of HNF4α and its target genes in Zucker diabetic fatty (ZDF) rats.

Gene	Organ	Treatment	Mean±SD	% of the control	p-value
**HNF4**α	Pancreas	Control	0.013±0.002		
		ZDF 9 months	0.008±0.004	61.5	**0.0494**
**PCK1**	Pancreas	Control	0.857±0.849		
		ZDF 9 months	0.365±0.541	42.6	**0.0191**
**INS1**	Pancreas	Control	0.146±0.076		
		ZDF 9 months	0.109±0.217	74.5	**0.0126**
**INS2**	Pancreas	Control	0.960±0.487		
		ZDF 9 months	0.456±0.871	47.5	**0.0052**
**HNF1**α	Liver	Control	1.379±0.611		
		ZDF 14 weeks	0.835±0.365	60.6	**0.0494**
**HNF4α**	Liver	Control	1.180±0.330		
		ZDF 14 weeks	0.694±0.228	58.8	**0.0015**

Gene expression was measured by real-time qRT-PCR in 14 weeks and 9 months old ZDF rats and lean controls (n = 10 animals, respectively) and was determined relative to expression of cyclophilin, which served as a housekeeping gene. Gene expression in control rats was set to 100 and values for ZDF rats represent transcript abundance relative to control. Non-parametric Mann-Whitney-U-Test was used to compare ZDF and control groups. Results are considered significant at p<0.05 (gene names and p-values in bold). Gene expression of HNF4α in the liver of this cohort of ZDF rats has been previously reported [43].

**Figure 2:**
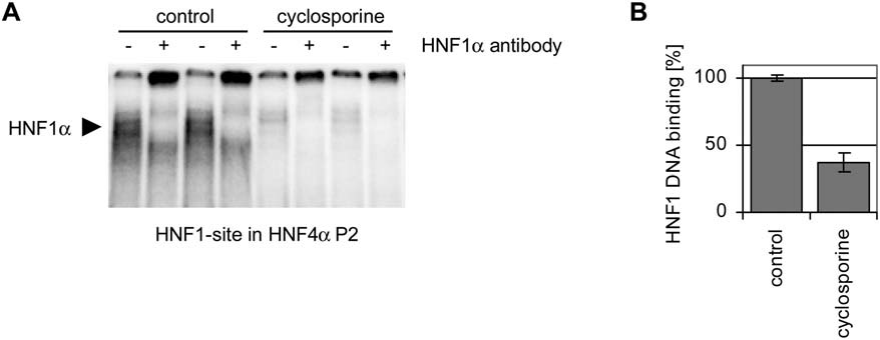
Cyclosporine inhibits HNF1α to the P2 promoter of HNF4α. (A) Electrophoretic mobility shift assays with 2,5 µg Caco-2 cell nuclear extract [control or cyclosporine treatment, 25 µM (30 µg/ml) for 72 h] and ^32^P labeled oligonucleotides to probe for DNA binding to the HNF1α binding-site within the HNF4α P2 promoter (HNF1-site in HNF4α P2). In EMSA supershift assays an antibody directed against HNF1α was added. Control and treated probes were run on same gels. (B) Dried EMSA gels were analyzed with a Molecular Imager (BioRad) using the Quantity One software (BioRad). HNF1α binding of control extracts was set to 100% and inhibition of binding after treatment with cyclosporine [25 µM (30 µg/ml) for 72 h] was quantified.

To further probe for HNF4α and HNF1α function we cultured the human intestinal cell line Caco-2. This cell line enables mechanistic studies with HNF4α protein expression being comparable to its expression levels in the liver [31]. In cell culture experiments we analyzed the effect of cyclosporine on HNF4α and HNF1α expression and activity. HNF4α gene and protein expression (Table 2, Fig 1A) as well as HNF1α protein expression (Fig 1B) was significantly repressed after treatment of Caco-2 cells with 25 µM (30 µg/ml) cyclosporine for 72 h, but HNF1α gene expression remained unchanged (Table 2). For comparison actin western blotting was used as housekeeping protein (Fig. 1C). Additionally, we investigated expression of genes coding for glucose metabolism, i.e. apolipoprotein C2 (*ApoC2*), aldehyde dehydrogenase 2 (*ALDH2*), phosphoenolpyruvate carboxykinase 1 (*PCK1*), glycerol kinase (*GK*), pyruvate kinase (*PKLR*) and aldolase B (*ALDOB*), and found *ApoC2*, *GK*, *PKLR* and *ALDOB* transcripts to be significantly repressed (Table 2). We further studied the ability of HNF4α to bind to promoter sequences of *HNF1α*, *ApoC2*, *GK*, *PKLR*, *ALDOB*, and *INS2* by EMSA supershift assays. As shown in Fig. 1D we observed strong binding of nuclear extracts of untreated cell cultures to all cognate recognition sites. Addition of a specific HNF4α antibody shifted the band, therefore providing clear evidence for the specificity of the assay. Strikingly, cyclosporine reduced binding of HNF4α to all EMSA probes employed to approximately 20% when compared with untreated cell cultures (Fig 1D, 1E). Binding activity of HNF1α to its recognition site in the pancreas specific P2 promoter of *HNF4α* was reduced as well (Fig. 2A, 2B), but treatment with equimolar concentrations of the calcineurin inhibitor tacrolimus did not influence HNF4α gene expression (Table 3).

**Table 2:** Regulation of gene expression in Caco-2 cells after cyclosporine treatment.

Gene	Treatment	Mean±SD	% of the control	p-value
**HNF4α**	Control	0.698±0.060		
	Cyclosporine	0.267±0.008	38.3	**0.0495**
HNF1α	Control	0.910±0.094		
	Cyclosporine	0.968±0.069	106.4	0.5127
**ApoC2**	Control	1.105±0.066		
	Cyclosporine	0.601±0.251	54.4	**0.0495**
ALDH2	Control	0.503±0.167		
	Cyclosporine	0.539±0.063	107.2	0.8273
PCK1	Control	1.056±0.136		
	Cyclosporine	0.840±0.266	79.5	0.2753
**GK**	Control	0.647±0.231		
	Cyclosporine	0.251±0.098	38.8	**0.0495**
**PKLR**	Control	0.784±0.229		
	Cyclosporine	0.290±0.126	37.0	**0.0495**
**ALDOB**	Control	0.204±0.067		
	Cyclosporine	0.035±0.025	17.2	**0.0495**
NFATc1	Control	0.449±0.236		
	Cyclosporine	0.498±0.065	110.9	0.5127
NFATc2	Control	0.655±0.193		
	Cyclosporine	0.495±0.196	75.6	0.5127
NFATc3	Control	1.154±0.260		
	Cyclosporine	0.938±0.134	81.3	0.2752
NFATc4	Control	0.974±0.251		
	Cyclosporine	0.793±0.151	81.4	0.2752
Calcineurin	Control	1.234±0.222		
	Cyclosporine	0.906±0.533	73.3	0.5127

Gene expression was measured by RT-PCR in Caco-2 cells 72 h after treatment with 25 µM (30 µg/ml) cyclosporine (n = 3, respectively) and was determined relative to expression of mitATPase6, which served as a housekeeping gene. Gene expression in untreated Caco-2 cells was set to 100 and values for cyclosporine treatment represent transcript abundance relative to control. Non-parametric Mann-Whitney-U-Test was used to compare cyclosporine treated and control groups. Results are considered significant at p<0.05 (gene names and p-values in bold).

**Table 3:** HNF4α gene expression in Caco-2 cells after tacrolimus treatment.

Gene	Treatment	Mean±SD	p-value
HNF4α	Control	1.373±0.347	
	Tacrolimus	1.166±0.127	0.5127

Gene expression was measured by real time qRT-PCR in Caco-2 cells 72 h after treatment with 25 µM (20 µg/ml) tacrolimus (Astellas Pharma GmbH, Munich, Germany) (n = 3, respectively) and was determined relative to expression of mitATPase6, which served as a housekeeping gene. Non-parametric Mann-Whitney-U-Test was used to compare tacrolimus treated and control groups. Results are considered significant at p<0.05.

**Figure 3:**
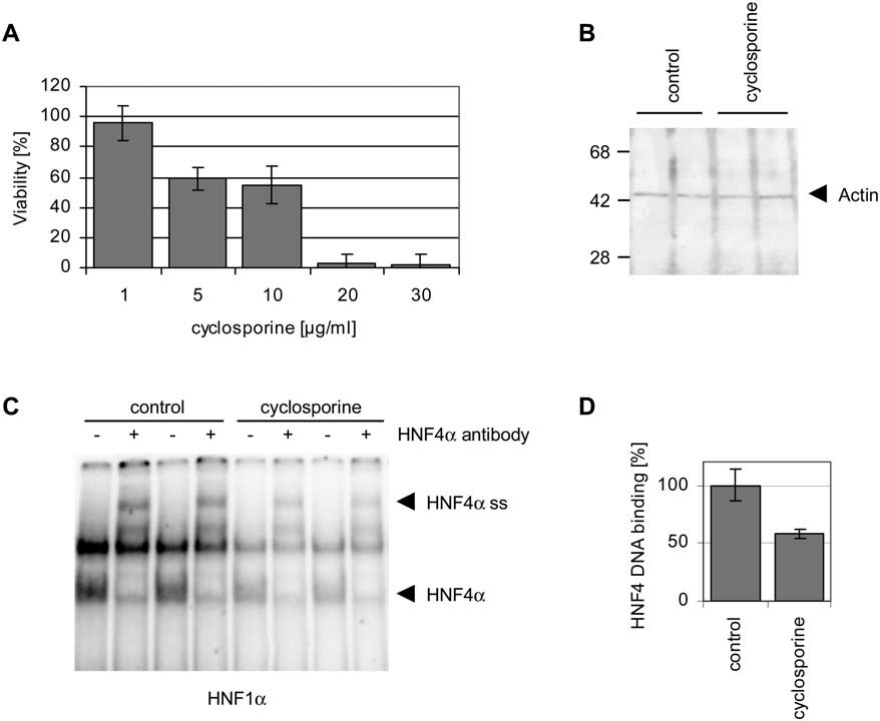
Cyclosporine inhibits binding of HNF4α at targeted gene promoters in INS-1E cells. (A) Cell viability of INS-1E cells after multiple treatments with cyclosporine for 72 h. (B) Actin western blotting of 10 µg INS-1E cell nuclear extracts [control or cyclopsorin treatment, 10 µg/mL (8.3 µM) for 72 h]. (C) Electrophoretic mobility shift assays with 20 µg INS-1E cell nuclear extract [control or cyclosporine treatment, 8.3 µM (10 µg/ml) for 72 h] and ^32^P labeled oligonucleotide to probe for DNA binding to the HNF4α binding-site within the promoter of HNF1α (HNF1α). In EMSA supershift assays an antibody directed against HNF4α (+) was added. Shifted (HNF4α) and supershifted bands (HNF4α ss) were marked. (D) Dried EMSA gels were analyzed with a Molecular Imager (BioRad) using the Quantity One software (BioRad). HNF4α binding of control extracts was set to 100% and inhibition of binding after treatment with cyclosporine [8.3 µM (10 µg/ml) for 72 h] was quantified.

To further confirm cyclosporine mediated dysregulation of HNF4α we analyzed different rat and mouse beta cell lines, i.e. INS-1E, RINm5F and MIN6 cells, for its *HNF4α* expression. INS-1E cells express *HNF4α* more abundantly and therefore were used for subsequent experiments (Table 4). As INS-1E cells are much more sensitive to the cyclosporine induced toxicity effects than Caco-2 cells, cell viability was tested at different cyclosporine concentrations. Treatment of INS-1E cells with 8.3 µM (10 µg/ml) cyclosporine (one third of the concentration used for Caco-2 cells) resulted in a 55% viability (Fig. 3A). In western blotting experiments actin served as a housekeeping protein, which we found to be constantly expressed (Fig. 3B). HNF4α protein expression of INS-1E cells is much lower than in liver [32]. In nuclear protein extracts HNF4α expression was below the limit of detection but its gene expression was unchanged (Table 5). Nonetheless, HNF4α DNA binding activity could be assayed for in EMSA supershift assays and was significantly reduced to 58% after treatment with 8.3 µM (10 µg/ml) cyclosporine (Fig. 3C, 3D). It is of considerable importance that the gene expression of the HNF4α target genes insulin1 (*INS1*) and insulin2 (*INS2*) was significantly repressed (Table 5).

**Table 4:** HNF4α gene expression in different beta cell lines.

Beta cell line	Species	Gene	% Expression
INS-1E	Rat	HNF4α	25.039±7.968
RIN-m5F	Rat	HNF4α	1.289±0.071
MIN6	Mouse	HNF4α	0.094±0.026

HNF4α gene expression was measured by real time qRT-PCR in INS-1E, Rin-m5F or MIN6 cells after 6 days in culture (n = 3, respectively). Gene expression was determined relative to expression of mitATPase6, which served as a housekeeping gene. Gene expression in untreated liver was set to 100% and values for gene expression in beta cells were calculated respectively.

**Table 5:** Regulation of gene expression in INS-1E cells after cyclosporine treatment.

Gene	Treatment	Mean±SD	% of the control	p-value
HNF4α	Control	0.849±0.308		
	Cyclosporine	0.984±0.066		0.5127
**INS1**	Control	0.128±0.003		
	Cyclosporine	0.087±0.004	68.0	**0.0495**
**INS2**	Control	1.076±0.237		
	Cyclosporine	0.335±0.039	31.3	**0.0495**

Gene expression was measured by real-time qRT-PCR in INS-1E cells 72 h after treatment with 8.3 µM (10 µg/ml) cyclosporine (n = 3, respectively) and was determined relative to expression of mitATPase6, which served as a housekeeping gene. Gene expression in untreated INS-1E cells was set to 100 and values for cyclosporine treatment represent transcript abundance relative to control. Non-parametric Mann-Whitney-U-Test was used to compare cyclosporine treated and control groups. Results are considered significant at p<0.05 (gene names and p-values in bold).

**Figure 4:**
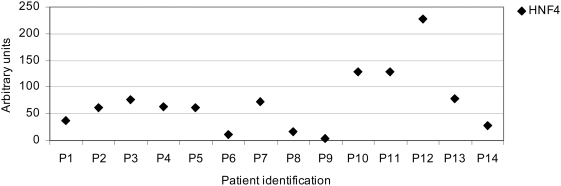
Gene expression of HNF4α in liver of human patients. Gene expression was determined by real-time qPCR in n = 14 patients. Characteristics of patients are given in Table 7.

**Figure 5:**
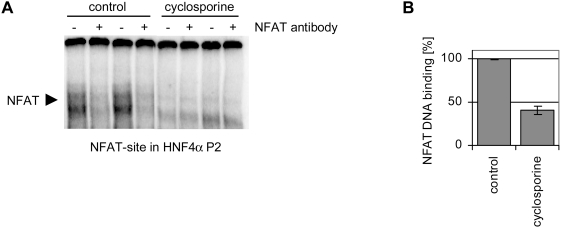
Cyclosporine inhibits NFAT binding to the P2 promoter of HNF4α. (A) Electrophoretic mobility shift assays with 2,5 µg Caco-2 cell nuclear extract [control or cyclosporine treatment, 25 µM (30 µg/ml) for 72 h] and ^32^P labeled oligonucleotides to probe for DNA binding to the NFAT binding site within the HNF4α P2 promoter (NFAT-site in HNF4α P2). In EMSA supershift assays an antibody directed against NFAT was added. Control and treated probes were run on same gels. (B) Dried EMSA gels were analyzed with a Molecular Imager (BioRad) using the Quantity One software (BioRad). NFAT binding of control extracts was set to 100% and inhibition of binding after treatment with cyclosporine [25 µM (30 µg/ml) for 72 h] was quantified.

Taken collectively, HNF4α and HNF1α expression and DNA-binding activity was repressed after cyclosporine treatment as was transcription of genes in the glucose and insulin signaling pathways targeted by HNF4α and HNF1α. Our study is the first report to determine a direct connection between cyclosporine treatment and activity of hepatic nuclear factors and our findings provide a molecular rational for PTDM observed in transplant patients. We suggest individual differences in the HNF4α gene and protein expression amongst patients to be of critical importance for the diabetogenic potential of cyclosporine. Indeed, on average 1/10 of cyclosporine treated patients develop PTDM. Consequently, repression of HNF4α by cyclosporine depends on the abundance of HNF4α protein. In Fig. 4 *HNF4α* gene expression in the liver of 14 patients was plotted; the data are scattered over a wide range. Likely, patients with low HNF4α and HNF1α protein would be at higher risk of developing cyclosporine induced PTDM. Specifically, cyclosporine binds to calcineurin and inhibits Ca^2+^-dependent serine / threonine phosphatase activity [33]. Normally this phosphatase dephosphorylates nuclear factor of activated T-cells (NFAT), which moves from the cytoplasm to the nucleus to associate with other proteins, thereby regulating expression of interleukin-2, granulocyte macrophage colony stimulating factor (GM-CSF), TNFα, IFNγ and other interleukins [34], [35]. Although inhibition of calcineurin results in immunosuppression, altering activity of NFAT will also impact regulation of INS1 gene transcription. Indeed, this factor is activated by calcineurin in response to increased Ca^2+^-levels [36]. Disruption of the NFAT/insulin pathway may contribute to the diabetogenic effects of cyclosporine as will be discussed below. Notably, Heit et al [37] reported the β-cell specific deletion of calcineurin to result in age-dependent diabetes, while conditional expression of activated NFAT reverted the diabetic phenotype in these mice. Furthermore, expression of genes critical for β-cell endocrine function e.g. HNF4α and HNF1α was increased in mice when NFATc1 was conditionally activated [37]. It is of considerable importance that NFAT cooperates with other transcription factors involved in insulin transcription such as PDX1, NEUROD1 and HNF4α. The evidence for this cooperation stems from chromatin immunoprecipitation assays [37]. The calcineurin/NFAT signaling appears to be essential for the regulation of pancreatic β-cell function; its cooperation with HNF4α could provide a molecular rational for cyclosporine induced PTDM [37]. HNF4α activity differs amongst cell types, in part due to use of alternate promoters. Whilst in hepatocytes the P1 promoter of *HNF4α* is primarily activated, the P2 promoter is specifically activated in pancreatic β-cells [17], [18] Indeed, P2 is exclusively expressed in INS-1E cells, see Table 6. In the study of Heit et al [37] binding of NFAT to the P1 promoter of *HNF4α* (NM_008261) was observed. The findings of Heit et al [37] are surprising as for normal β-cell function usage of the P2 promoter of *HNF4α* would have been expected. Notably, we observed NFAT binding at the human P2 promoter of *HNF4α* at position −461 to −450 upstream of the start site of transcription (see Material and Methods for sequence information). Furthermore, binding of NFAT to the *HNF4α* P2 promoter was reduced in response to cyclosporine treatment (Fig. 5A, 5B), but expression of members of the NFAT gene family (NFATc1, c2, c3, c4) and of calcineurin itself was unchanged after cyclosporine treatment of Caco-2 cells (Table 2). There is clear evidence for a role of NFAT in glucose/insulin homoeostasis [38]. NFAT signaling plays an essential role in the development of diabetes in calcineurin knock-out mice [37]. Taken collectively, we report a remarkable repression of HNF4α and HNF1α after cyclosporine treatment and propose cyclosporine to act through a calcineurin/NFAT dependent mechanism on these transcription factors. We further identified a novel NFAT binding site in the human *HNF4α* P2 promoter and report HNF4α activity and expression of genes of the glucose/insulin signaling pathway to be reduced in the pancreas of ZDF diabetic rats.

**Table 6:** HNF4α isoform expression in INS-1E cells.

HNF4α isoform	Mean±SD
HNF4αP1	0
HNF4αP2	418.18±225.99

HNF4α isoform expression was measured by real time qRT-PCR in INS-1E cells after 6 days in culture (n = 3, respectively). Gene expression was determined relative to expression of mitATPase6, which served as a housekeeping gene. Gene expression in rat liver served as positive control for HNF4αP1 expression, gene expression in rat pancreas served as positive control for HNF4αP2 expression.

**Table 7:** Patient characteristics.

Patient Identification	Sex	Age	Tissue	Information
P1	F	40	Healthy tissue from liver resection	Colorectal liver metastasis
P2	M	42		Colorectal liver metastasis
P3	F	48		Colorectal liver metastasis
P4	F	61		Colorectal liver metastasis
P5	F	61		Colorectal liver metastasis
P6	M	67		Hepatocellular carcinoma
P7	F	70		Hepatocellular carcinoma
P8	F	57		Hepatocellular carcinoma
P9	M	67		Hepatocellular carcinoma
P10	M	67		Liver metastasis, stomach cancer
P11	M	72		Liver metastasis, gastrointestinal stromal tumor
P12	M	69		Colorectal liver metastasis
P13	M	76		Hepatocellular carcinoma
P14	F	57		Epitheloidal angiolipoma

Patient material was used with a permission from the ethics committee of the Medical School Hannover, Germany.

In conclusion, cyclosporine repressed HNF4α/HNF1α expression, DNA-binding to targeted promoters and subsequent expression of genes involved in glucose metabolism and pancreatic β-cell function. We propose a molecular mechanism for PTDM based on dysregulation of HNF4α/HNF1α and of NFAT insulin signaling pathway targeted by cyclosporine.

## Materials and Methods

### Cell culture and cyclosporine treatment

Caco-2 cells, a human intestinal cell line derived from a colon adeno-carcinoma, were obtained from and cultivated as recommended by DSMZ (Deutsche Sammlung von Mikroorganismen und Zellkulturen GmbH, Braunschweig, Germany). INS-1E cells (rat beta cells derived from insulinomas) were kindly provided by C. Wollheim (University Medical Center, Geneva, Switzerland) [39], MIN6 cells (mouse beta cells transgenic for SV40 large T antigen) were kindly provided by J. Miyazaki (Osaka University Medical School, Japan) [40] and RIN-m5F cells (rat beta cells derived from islet cell tumor) were kindly provided by S. Lenzen (Medical School Hannover, Germany) [41]. Caco-2 cells were daily treated with 25 µM (30 µg/ml) and INS-1E cells with 8.3 µM (10 µg/ml) cyclosporine (Sandimmun, Novartis, Nürnberg, Germany) for 72 h. Treatment started at 40–50% confluence. Cell viability was analyzed in triplicate using a MTS cytotoxicity assay according to the manufacturers instructions (#G3582, Promega, Mannheim, Germany).

### Diabetic disease model

Pancreas (animals aged 9 months) and liver (animals aged 14 weeks) of fa/fa obese Zucker diabetic fatty (ZDF) rats and of +/fa lean nondiabetic control rats were kindly provided by W. Linz and H. Ruetten (Sanofi-Aventis, Frankfurt, Germany) [42]. Pancreatic mRNA degrades quickly, i.e. in less than 1 minute after tissue resection, therefore, pancreas was frozen immediately. All rats were male with mean body weight of 398.8±30.2 (obese) and 334.2±19.3 (lean) for 14 weeks aged animals and 403.8±35.7 (obese) and 463.3±30.3 (lean) for 9 months aged animals. Representative phenotype data (e.g. blood glucose, insulin) are provided in Niehof et al [43].

### Isolation of nuclear extracts, western blotting analysis and electrophoretic mobility shift assays

Nuclear extracts were isolated by the method of Dignam et al [44] with minor modifications as detailed previously [31]. Details for western blotting analysis and electrophoretic mobility shift assays were given in Niehof and Borlak, 2005 [31]. Antibodies directed against HNF4α (sc-6556), HNF1α (sc-6547), and Actin (sc-1616) were purchased from Santa Cruz Biotechnology (Heidelberg, Germany). Nuclear extracts were prepared mainly in triplicate and used as described in the figure legend. The antigen-antibody complexes were visualized using the enhanced chemiluminescence (ECL) detection system (PerkinElmer Life Sciences, Rodgau-Juegesheim, Germany). Light signal detection was done with the CCD camera Imager system Kodak IS 440 CF (Kodak, Biostep GmbH, Jahnsdorf, Germany) and quantification was performed using the Kodak 1D Image analysis software (version 3.5.). The oligonucleotides were purchased from MWG Biotech (Ebersberg/Muenchen, Germany) with the following sequences: AAG GCT GAA GTC CAA AGT TCA GTC CCT TC (HNF1α, NM_000545), TGT CTA GGC CAA AGT CCT GGC CA (ApoC2, apolipoprotein C2, NM_000483), GCT GCC TGC CAA AGG GCA GTA CT (GK, glycerol kinase, NM_203391), AGA TGA GGG CAG AGA GCA GGC CG (PKLR, pyruvate kinase, NM_000298), ACA AAA GTA CAA AGG TTA AAA GA (ALDOB, aldolase B, NM_000035), GAC AAA CAG CAA AGT CCA GGG GT (INS2, insulin 2, NM_019130), GAC TGG TTA CTC TTT AAC GTA TC (HNF1-site in HNF4α, NM_001030004), and CCC TCC TTT TTT CCT CTG CCC CT [NFAT-site (nuclear factor of activated T-cells) in HNF4α, NM_001030004] and were ^32^P-labeled. Super shift assays were done with HNF4α specific antibody (sc-6556x), HNF1α specific antibody (sc-6547x), and NFAT specific antibody (sc-1149x), all were purchased from Santa Cruz Biotechnology, Heidelberg, Germany and once again details are given in [31].

### RT-PCR and real-time semi-quantitative PCR

Total RNA was isolated using the nucleospin RNA Isolation Kit (Macherey-Nagel) according to the manufacturers recommendations. 4 µg total RNA from each sample was used for reverse transcription (Omniscript Reverse Transcriptase, Qiagen, Hilden, Germany). PCR was done in a mixture containing a cDNA equivalent to 25 ng of total RNA, 1 µM of each primer, 0.25 mM dNTP mixture, 0.625 U Thermostart-Taq (Abgene, Hamburg, Germany) and 1× PCR-buffer (Abgene, with 1.5 mM MgCl_2_) in a total volume of 20 µl. PCR-reactions were carried out with a thermocycler (T3, Biometra, Göttingen, Germany) with the following conditions: initial denaturation at 95°C for 15 min (Thermostart activation), denaturation at 94°C for 30 sec, annealing at different temperatures for 45 sec (see below), extension at 72°C for 45 sec, final extension at 74°C for 10 min. The following primer pairs were used: HNF4α (human, NM_000457), fwd: CTG CTC GGA GCC ACA AAG AGA TCC ATG, rev: ATC ATC TGC CAC GTG ATG CTC TGC A (50°C, 29cyc); HNF1α (human, NM_000545), fwd: TCT ACA ACT GGT TTG CCA ACC, rev: GGC TTC TGT ACT CAG CAG GC (50°C, 33cyc); ApoC2 (apolipoprotein C2) (human, NM_000483), fwd: CCT CCC AGC TCT GTT TCT TG, rev: GCT GCT GTG CTT TTG CTG TA (60°C, 38cyc); GK (glycerol kinase) (human, NM_203391), fwd: AGT CTC GAA CCC GAG GAT TT, rev: GTC ATG CAG CAA GTG GCT TA (55°C, 36cyc); PKLR (pyruvate kinase) (human, NM_000298), fwd: GTG GAG AGC TTT GCA GGT TC, rev: GCC GAT TTT CTG GAC CAC TA (55°C, 36cyc); ALDOB (aldolase B) (human, NM_000035), fwd: GCT CTC CAC CGT ACT GTT CC, rev: CCA GAA GAA CCC GTG TGA AC (50°c, 38cyc); ALDH2 (aldehyde dehydrogenase 2) (human, NM_000690), fwd: TGA AGG GGA CAA GGA AGA TG, rev: ACA GGT TCA TGG CGT GTG TA (58°C, 33cyc); PCK1 (phosphoenolpyruvate carboxykinase) (human, NM_002591), fwd: TCA GGC GGC TGA AGA AGT AT, rev: ACG TAG GGT GAA TCC GTC AG (60°C, 40cyc); NFAT (nuclear factor of activated T-cells) c1 (human, NM_172389), fwd: AGA AAG CGA AGC CAG TAC CA, rev: GAG AAA GGT CGT GGA GCT TG (60°C, 40cyc); NFATc2 (human, NM_012340), fwd: CAC GGG GCA GAA CTT TAC AT, rev: GCA GAT CAG AGT GGG GTC AT (60°C, 32cyc); NFATc3 (human, NM_173164), fwd: CTC AGT GGG AGG TAG AAG GG, rev: TGT TTG TGG GAT GGA GCA AA (60°C, 34cyc); NFATc4 (human, NM_004554), fwd: CCA GAC TCC AAG GTG GTG TT, rev: CTG GGT GGT GAG AAG TCC AT (60°C, 38cyc); calcineurin (PPP3R1) (human, NM_000945), fwd: CTC ACA CTT TGA TGC GGA TG, rev: TTG TTC CCC ACC ATC ATC TT (50°C, 32cyc); mitATPase (human, NC_001807), fwd: CTA AAG GAC GAA CCT GA, rev: TGG CCT GCA GTA ATG TT (55°C, 25cyc).

Real-time RT-PCR measurement was done with the Lightcycler (Roche Diagnostics, Mannheim, Germany) with the following conditions: denaturation at 94°C for 120 sec, annealing at different temperatures for 8 sec (see below), extension at 72°C for different times (see below), fluorescence at different temperatures (see below). The PCR reaction was stopped after a total of 40–45 cycles and at the end of each extension phase, fluorescence was observed and used for quantification within the linear range of amplification. Exact quantification was achieved by serial dilution with cDNA produced from total RNA extracts using 1∶5 dilution steps. Gene expression levels were normalized to cyclophilin, which was found to be stably expressed. The following primer pairs were used: HNF4α (rat, NM_022180), fwd: GCC TGC CTC AAA GCC ATC AT, rev: GAC CCT CCA AGC AGC ATC TC (55°C, 11 sec, 88°C); HNF4αP1 (rat, D10554), fwd: AAA TGT GCA GGT GTT GAC CA, rev: CAC GCT CCT CCT GAA GAA TC (60°C, 7 sec, 87°C); HNF4αP2 (rat, AF329936), fwd: CTC CAG TGG CGA GTC CTT AT, rev: TCA CGC TCC TCC TGA AGA AT (60°C, 7 sec, 87°C); HNF4α (mouse, NM_008261), fwd: ACA CGT CCC CAT CTG AAG, rev: CTT CCT TCT TCA TGC CAG (68°C, 12 sec, 86°C); PCK1 (rat, NM_198780), fwd: ACG CCA TTA AGA CCA TCC AG, rev: TTC GTA GAC AAG GGG GAC AC (60°C, 13 sec, 87°C); INS1 (rat, NM_019129), fwd: AGA CCA TCA GCA AGC AGG TC, rev: CCA GTT GGT AGA GGG AGC AG (68°C, 14 sec, 88°C); INS2 (rat, NM_019130), fwd: CAG CAC CTT TGT GGT TCT CA, rev: CAG TGC CAA GGT CTG AAG GT (60°C, 7 sec, 87°C); cyclophilin rat, NM_017101), fwd: TTT CGT GCT CTG AGC ACT GG, rev: CTT GCC ATT CCT GGA CCC AA (55°C, 15 sec, 82°C); mitATPase (rat, NC_001807), fwd: CTA AAG GAC GAA CCT GA, rev: TGG CCT GCA GTA ATG TT (55 C°, 13 sec, 83°C).

### Statistical analysis

All values are expressed as mean±standard deviation. To determine significance between two groups, comparison was made using the non-parametric two-tailed Mann-Whitney-U-Test. Therefore, Statistica software, version 7.1 (StatSoft) was used. The results are considered significant when the p value was less than 0.05.
